# Ammonium sorption and ammonia inhibition of nitrite-oxidizing bacteria explain contrasting soil N_2_O production

**DOI:** 10.1038/srep12153

**Published:** 2015-07-16

**Authors:** Rodney T. Venterea, Timothy J. Clough, Jeffrey A. Coulter, Florence Breuillin-Sessoms

**Affiliations:** 1 USDA-ARS, Soil and Water Management Research Unit, St. Paul, MN 55108; 2 Dep. of Soil, Water, and Climate, Univ. of Minnesota, St. Paul, MN 55108; 3 Faculty of Agriculture and Life Science, Lincoln Univ., PO Box 85084, Lincoln 7647, Canterbury, New Zealand; 4 Dep. of Agronomy and Plant Genetics, Univ. of Minnesota, St. Paul, MN 55108; 5 Biotechnology Institute, College of Biological Sciences, Univ. of Minnesota, St. Paul, MN 55108.

## Abstract

Better understanding of process controls over nitrous oxide (N_2_O) production in urine-impacted ‘hot spots’ and fertilizer bands is needed to improve mitigation strategies and emission models. Following amendment with bovine (*Bos taurus*) urine (Bu) or urea (Ur), we measured inorganic N, pH, N_2_O, and genes associated with nitrification in two soils (‘*L*’ and ‘*W*’) having similar texture, pH, C, and C/N ratio. Solution-phase ammonia (*sl*NH_3_) was also calculated accounting for non-linear ammonium (NH_4_^+^) sorption capacities (ASC). Soil *W* displayed greater nitrification rates and nitrate (NO_3_^−^) levels than soil *L*, but was more resistant to nitrite (NO_2_^−^) accumulation and produced two to ten times less N_2_O than soil *L*. Genes associated with NO_2_^−^ oxidation (*nxr*A) increased substantially in soil *W* but remained static in soil *L*. Soil NO_2_^−^ was strongly correlated with N_2_O production, and cumulative (*c-*) *sl*NH_3_ explained 87% of the variance in *c-*NO_2_^−^. Differences between soils were explained by greater *sl*NH_3_ in soil *L* which inhibited NO_2_^−^ oxidization leading to greater NO_2_^−^ levels and N_2_O production. This is the first study to correlate the dynamics of soil *sl*NH_3_, NO_2_^−^, N_2_O and nitrifier genes, and the first to show how ASC can regulate NO_2_^−^ levels and N_2_O production.

Better understanding of biochemical process controls over soil N_2_O production is needed for improving N_2_O mitigation strategies and emissions models. Incorporation of improved process-related information into models will help improve emissions assessments at field, regional and global scales^1^,^2^. In cattle grazing systems, urine deposition events typically result in localized N loads equivalent to 1000 kg N ha^−1,^^3^, and can create ‘hot spots’ for elevated N_2_O emissions^4^. Similarly, N fertilizers applied in concentrated bands result in localized inorganic N concentrations ranging from several hundred to more than 2000 μg N g^−1,^^5^,^6^, and can result in substantially greater N_2_O emissions compared to uniformly applied fertilizer^7^,^8^,^9^. Thus, understanding the processes mediating N_2_O production within these concentrated zones is critical. Urine deposition and Ur application can cause elevated soil NO_2_^−^ levels which in turn promote elevated N_2_O production^9^,^10^,^11^. Accumulation of NO_2_^−^ is presumed to occur due to a sequence of chemical and microbial responses. Hydrolysis of Ur results in localized increases in both soil pH and NH_4_^+^ which together promote the formation of free NH_3_^12^. Both groups of autotrophic nitrifying bacteria, i.e., the NH_3_-oxidizing bacteria (AOB) and the NO_2_^−^ oxidizing bacteria (NOB), are sensitive to NH_3_ toxicity, but it is generally believed that NOB are more sensitive than AOB^11^,^12^,^13^,^14^; thus, soil NO_2_^−^ accumulates in the presence of sufficiently high NH_3_ levels because NOB are unable to fully process the NO_2_^−^ produced by AOB.

However, beyond this rather general understanding of soil NO_2_^-^ dynamics, little is known about specific soil properties that regulate NO_2_^−^ accumulation. While related processes have been well-studied in wastewater systems^14^, the presence of soil surface-solution interactions and other factors complicate our understanding in soils. For example, the partitioning of NH_4_^+^ between soil surfaces and solution could regulate solution-phase levels and therefore influence nitrifier activity and NH_3_ toxicity^15^. Simultaneous quantification of genes associated with activities of NOB, AOB and NH_4_^+^ oxidizing archaea in soil following amendment with N has been reported in a few studies^16^,^17^,^18^, but more data are needed to understand the role of nitrifier responses in regulating NO_2_^−^ and N_2_O dynamics. Limited understanding of these and other factors limits our ability to predict NO_2_^−^ dynamics for a particular soil, management practice, or climate condition, and our ability to predict N_2_O emissions resulting from NO_2_^−^ transformations^19^,^20^.

In preliminary experiments, we observed that two soils collected from grazed fields in New Zealand, while having similar texture, pH, C content and C/N ratio, displayed substantially different N_2_O production rates when amended with Ur. Our general hypothesis was that differences in NO_2_^−^ dynamics were responsible for the contrasting N_2_O production. In this study, we conducted a series of experiments designed to elucidate controls over NO_2_^−^ and N_2_O production under conditions representative of concentrated BU patches or Ur bands, and to explain the differences in N_2_O production between these soils.

## Results

### Ammonium sorption

**Figure 1:**
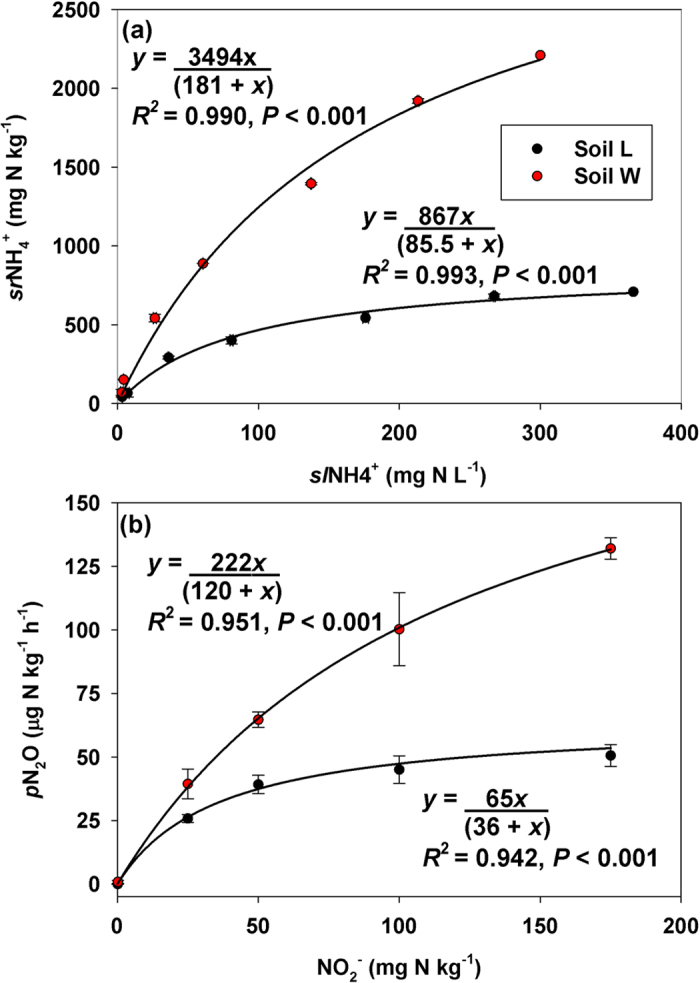
Ammonium sorption capacity (ACS) and potential N_2_O production (*p*N_2_O). (**a**) ASC results showing equilibrium NH_4_^+^ concentrations in sorbed-phase (*sr*NH_4_^+^) versus solution-phase (*sl*NH_4_^+^) and (**b**) *p*N_2_O following addition of nitrite (NO_2_^−^) at 85% field capacity for soils *L* and *W*. Symbols are means with vertical standard error bars and lines are regression curves based on replicated data (in form of Eq. (1)). Horizontal error bars are displayed in (**a**) but are barely visible.

Ammonium sorption, determined in batch equilibrium experiments and modeled using Eq. 1, was significantly greater in soil *W*, which sorbed more NH_4_^+^ from solution compared to soil *L* (Fig. 1a). The modeled sorption parameters μ and *K* in soil *W* were four and two times greater, respectively, than in soil *L* (Fig. 1a).

### Nitrite addition experiments

Soil *W* produced more N_2_O after amendment with NO_2_^−^ compared to soil *L*. The potential N_2_O production rate (*p*N_2_O) was well-described by models in the form of Eq. (1) with sorbed-phase NH_4_^+^ (*sr*NH_4_^+^) replaced by potential N_2_O production rate (*p*N_2_O), and with solution phase NH_4_^+^ (*sl*NH_4_^+^) replaced by NO_2_^−^ concentration (Fig. 1b). The modeled μ and *K* parameters in soil *W* were each approximately three times greater than in soil *L*.

### Microcosm experiments

#### Series 1 - Effect of BU addition rate at 85% of field capacity (FC)

**Figure 2:**
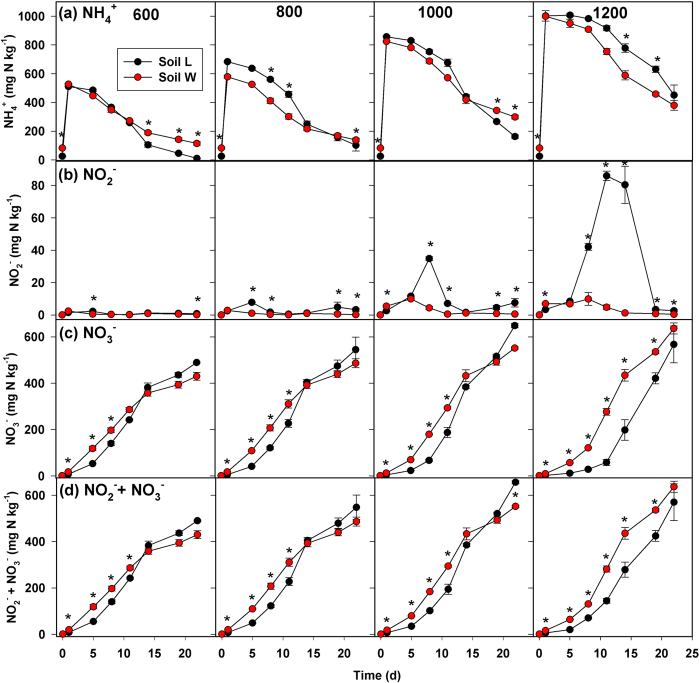
Results of Series 1 microcosm experiment. Concentrations of extractable (**a**) NH_4_^+^, (**b**) NO_2_^−^, (**c**) NO_3_^−^ and (d) NO_2_^−^ + NO_3_^−^ following addition of bovine urine at 600, 800, 1000 and 1200 mg N kg^−1^ at 85% field capacity. Asterisks indicate significant differences between soils at *P* < 0.05 for a given sampling date.

**Figure 3:**
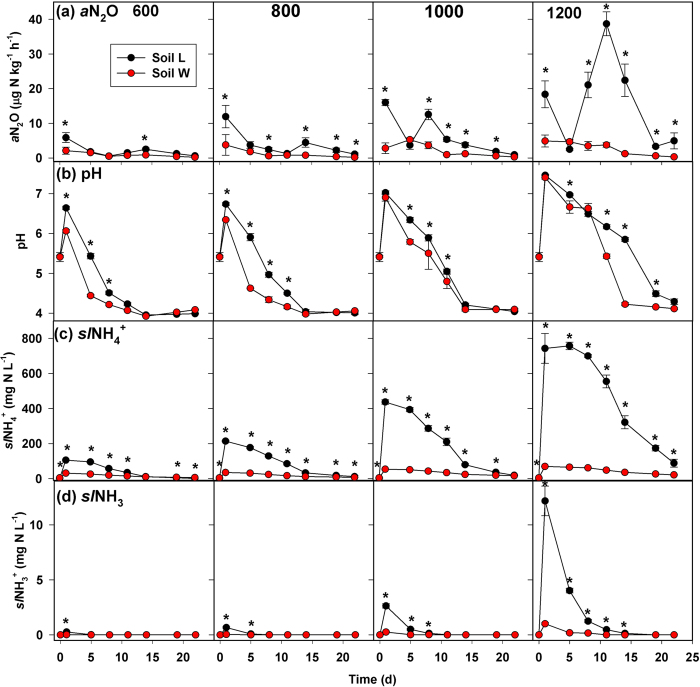
Results of Series 1 microcosm experiment (continued). (**a**) Actual N_2_O production rate (aN_2_O), (**b**) pH, and theoretical concentrations of solution-phase (**c**) ammonium (*sl*NH_4_^+^) and (**d**) ammonia (*sl*NH_3_) following addition of bovine urine at 600, 800, 1000 and 1200 mg N kg^−1^ at 85% field capacity. Asterisks indicate significant differences between soils at *P* < 0.05 for a given sampling date.

There were significant soil-by-BU addition rate-by-time interaction effects on all point-in-time (Figs 2, 3) and cumulative variables (Table 1). Most notably for point-in-time concentrations, soil *L* had greater NO_2_^−^ and actual N_2_O production rate (*a*N_2_O) compared to *W* on at least two sampling dates at all N rates, and the frequency and magnitude of significant differences in NO_2_^−^ and *a*N_2_O by soil increased with increasing BU addition (Figs 2b, 3a). Following BU addition, both soils showed a similar temporal pattern of increasing total extractable NH_4_^+^ (*t*NH_4_^+^) followed by decreasing *t*NH_4_^+^ (Fig. 2a). Differences in *t*NH_4_^+^ by soil were not observed until at least Day 8, and the timing and direction of significant differences varied by BU addition rate. Soil *W* had more nitrate (NO_3_^−^) and (NO_2_^−^+NO_3_^−^) present compared to soil *L* during at least the first 8 d, and the magnitude and duration of significant differences in NO_3_^−^ by soil increased with increasing BU addition (Figs 2c–d). Soil *L* consistently produced more N_2_O on Day 1 compared to *W*. Soil pH, *sl*NH_4_^+^ and *sl*NH_3_ were frequently greater in soil *L* compared to *W* (Figs 3b–d). Most notably for cumulative indices (Table 1), soil *L* had consistently greater *c-*NO_2_^−^, *c-sl*NH_4_^+^, *c-*NH_3_, and *c-a*N_2_O compared to *W* at all BU addition rates. During the first 5 d, soil *W* had greater NO_2_^−^+NO_3_^−^ accumulation rate (NAR) compared to soil *L* at all BU addition rates, and both soils showed reduced NAR at N ≥ 1000 mg N kg^−1^ compared to N < 1000 mg N kg^−1^ (Table S1). During Days 5 through 11, significant differences in NAR by soil were only present at 1000 and 1200 mg N kg^−1^. During Days 11 through 19, soil *L* had greater NAR compared to *W* at all BU addition rates except 1200 mg N kg^−1^.

#### Series 2 - Effect of soil water content with 1000 mg N kg^−1^ of BU added

**Figure 4:**
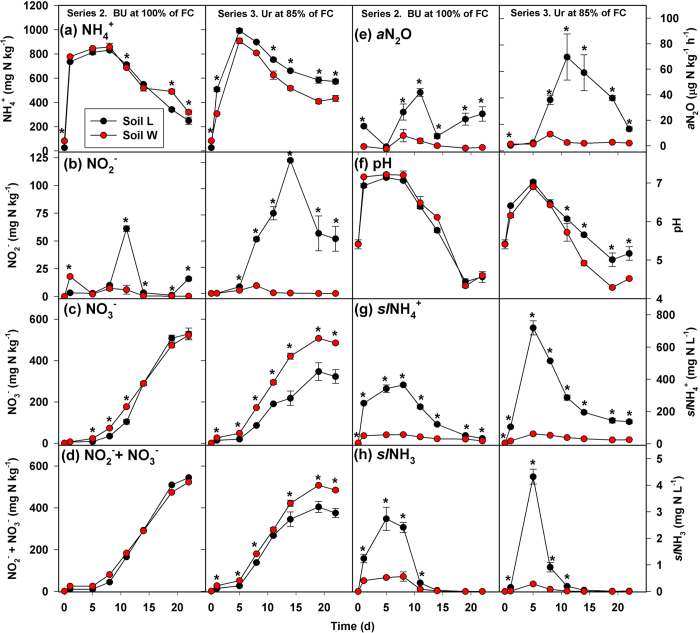
Results of Series 2 and 3 microcosm experiments. Concentrations of extractable (**a**) NH_4_^+^, (**b**) NO_2_^−^, (**c**) NO_3_^−^ and (**d**) NO_2_^−^ + NO_3_^−^, (**e**) actual N_2_O production rate (*a*N_2_O), (**f**) pH and theoretical concentrations of solution-phase (**g**) ammonium (*sl*NH_4_^+^) and (**h**) ammonia (*sl*NH_3_) in Series 2 (left-hand plates for each variable) and Series 3 (right-hand plates). Series 2 used bovine urine (BU) at 1000 mg N kg^−1^ with soils at 100% of field capacity (FC), and Series 3 used urea (Ur) at 1000 mg N kg^−1^ with soils at 85% of FC. Asterisks indicate significant differences between soils at *P* < 0.05 for a given sampling date.

There were significant soil-by-water content-by-time interaction effects on point-in-time concentrations of *t*NH_4_^+^, NO_2_^−^, NO_3_^−^, *a*N_2_O, *sl*NH_4_^+^, and *sl*NH_3_. Differences by soil in the 100% of FC treatment were similar to differences by soil in the 85% of FC treatment with 1000 mg N kg^−1^, except for pH and NO_2_^−^+NO_3_^−^ where no differences were observed (Fig. 4). For cumulative indices, there were significant soil-by-water content-by-time interaction effects on *c-t*NH_4_^+^, *c-*(NO_2_^−^ + NO_3_^−^), *c-sl*NH_4_^+^ and *c-a*N_2_O (Table S2). Differences in *c-a*N_2_O by soil were similar at 85% and 100% of FC, but both soils had greater *c-a*N_2_O at 100% compared to 85% of FC. Across both soils, *c-*NO_2_^−^ and *c-sl*NH_3_ were greater at 100% compared to 85% of FC, while *c-*NO_3_^-^ and *c-*H^+^ were greater at 85% compared to 100% of FC (Table S3). Across both water content treatments, *c-*NO_2_^−^ was greater in soil *L* compared to *W*.

#### Series 3- Effect of BU versus Ur added at 1000 ug N g^−1^ at 85% of FC

There were significant soil-by-N source-by-time interaction effects on all point-in-time variables, and differences by soil following Ur addition were generally consistent with differences following BU addition at 1000 mg N kg^−1^ (Fig. 4). However, differences by soil tended to be more consistent and/or to persist longer with Ur compared to BU. Also, *t*NH_4_^+^ and pH each took longer to reach their maximum values and did not decrease as rapidly with Ur compared to BU. Cumulative *t*NH_4_^+^, *c-*N_2_O, and *c-sl*NH_3_ were greater with Ur, while *c-*H^+^ was lower with Ur, compared with BU, in both soils (Table S4). Cumulative NO_2_^−^ was more than five times greater with Ur compared to BU for soil *L*.

**Figure 5:**
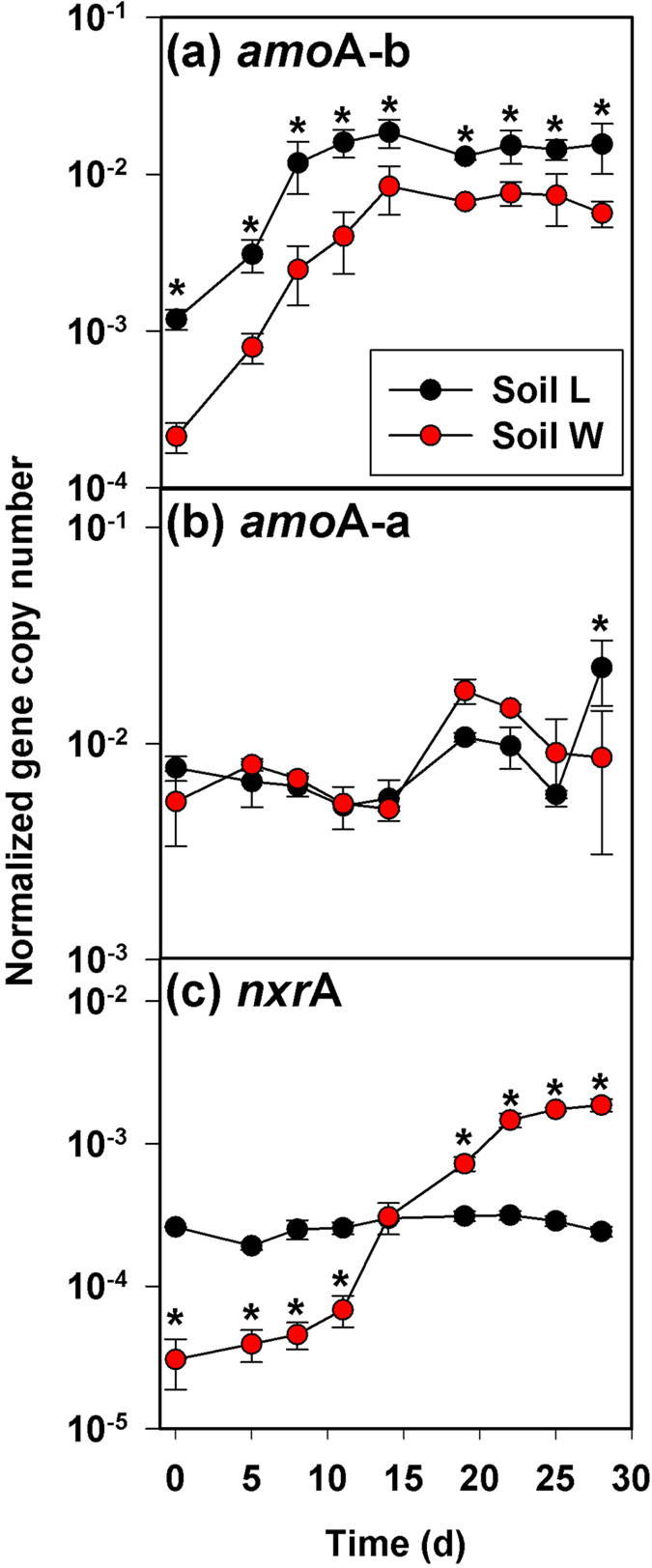
Gene copy abundances in Series 3 microcosm experiment. (**a**) *amo*A-b, (**a**) *amo*A-a, and (**c**) *nxr*A following addition of Ur at 1000 mg N kg^−1^ soil with soils at 85% of FC. Asterisks indicate significant differences between soils at *P* < 0.05. Normalized gene abundances are expressed relative to the number of copies of prokaryotic (bacteria + archaea) 16S rRNA genes in each sample^47^.

Gene copies of bacterial ammonia monooxygenase (*amo*A-b) associated with AOB were greater in soil *L* compared to *W*, but increased over time in a similar manner in both soils until Day 14, after which the abundances did not change (Fig. 5). There was a significant soil-by-time interaction effect on gene copies of archaeal ammonia monooxygenase (*amo*A-a), but a difference by soil was observed only on the final sampling date (Fig. 5b). In both soils, *amo*A-a numbers did not change until after Day 14, corresponding to the cessation of any increases in *amo*A-b. Most notable was a significant soil-by-time interaction effect on gene copies of bacterial nitrite oxidoreductase (*nxr*A) associated with NOB (*P* < 0.001), which were initially lower in soil *W* than *L*, and subsequently increased in soil *W* by a factor of 60 while remaining static in soil *L* (Fig. 5c).

#### Correlation and regression analyses

**Figure 6:**
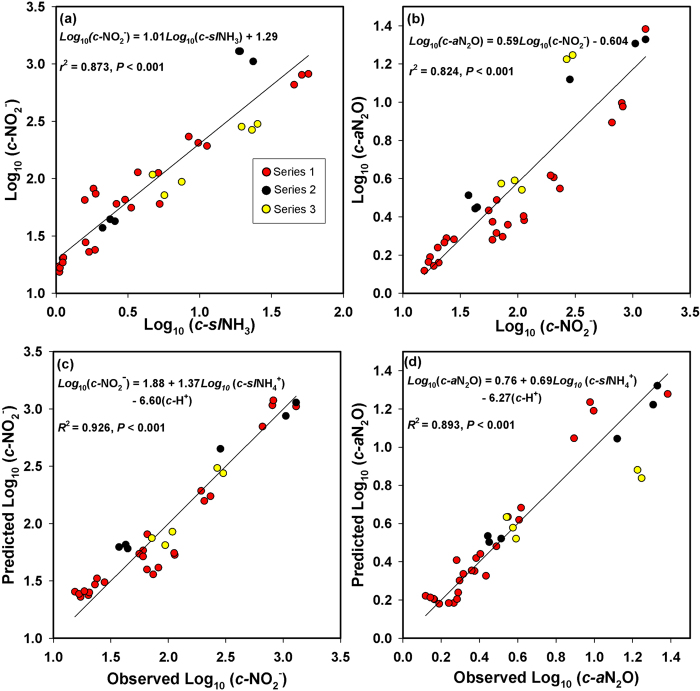
Regression results. Single-factor regression models of (**a**) cumulative nitrite (*c-*NO_2_^−^) versus cumulative solution-phase ammonia (*c-sl*NH_3_) and (**b**) cumulative actual N_2_O production (*c-a*N_2_O) versus *c-*NO_2_^−^ with regression lines, and multiple regression models describing (**c**) *c-*NO_2_^−^ and (d) *c-a*N_2_O as functions of cumulative solution-phase ammonium (*c-sl*NH_4_^+^) and cumulative acidity (*c-*H^+^) with 1:1 lines, for all microcosm data (Series 1–3).

Soil NO_2_^−^ was positively correlated with *t*NH_4_^+^, *sl*NH_4_^+^ and *sl*NH_3_, and *c-*NO_2_^−^ was positively correlated with *c-t*NH_4_^+^, *c-sl*NH_4_^+^ and *c-sl*NH_3_ (Table S5). Across all experiments (Series 1-3), *c-*NO_2_^−^ was most strongly correlated with *c-sl*NH_3_, which explained 87% of the total variance in *c-*NO_2_^−^ (Fig. 6a). Soil NO_2_^−^ was also positively correlated with *a*N_2_O, and *c-*NO_2_^−^ was positively correlated with *c-a*N_2_O. Across all experiments, *c-*NO_2_^−^ explained 82% of the total variance in *c-a*N_2_O (Fig. 6b). Soil NO_3_^−^ and (NO_2_^−^+NO_3_^−^) tended to be negatively correlated with N_2_O (Table S5). Multiple regression models with *c-sl*NH_4_^+^ and *c-*H^+^ as independent variables explained 93 and 89% of the variance in *c-*NO_2_^−^ and *c-a*N_2_O, respectively (Figs 5c,d).

## Discussion

**Figure 7:**
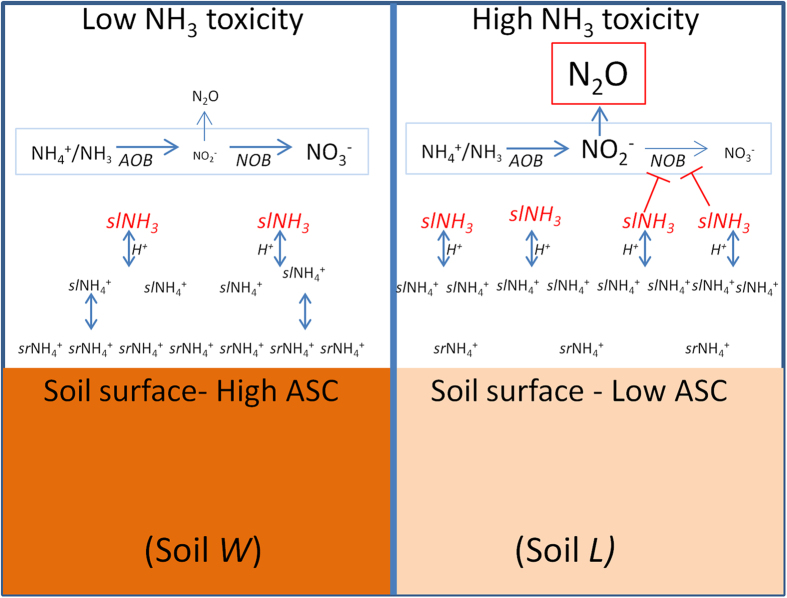
Conceptual schematic. Decreased ammonium (NH_4_^+^) sorption capacity (ASC) results in increased ratio between solution-phase (*sl*) and sorbed-phase (*sr*) NH_4_^+^, which increases the potential formation of free ammonia (*sl*NH_3_). When *sl*NH_3_ differentially inhibits nitrite (NO_2_^−^) oxidizing bacteria (NOB) to a greater extent than NH_3_ ammonia-oxidizing bacteria (AOB), NO_2_^−^ accumulates, leading to increased NO_2_^−^-driven N_2_O production in the low ASC soil.

This is the first study to correlate the dynamics of *sl*NH_3_, NO_2_^−^, N_2_O and nitrifier genes in incubating soil. The strong relationship (*r*^2^ = 0.87) between *c-sl*NH_3_ and *c-*NO_2_^−^ suggests that NH_3_ toxicity acting more strongly on NOB than AOB, and more strongly in soil *L* than in soil *W*, was responsible for the contrasting NO_2_^−^ and N_2_O dynamics in the two soils^11^,^12^,^13^,^14^. The greater N_2_O production in soil *L* appeared to be driven by a greater NO_2_^−^ accumulation which in turn resulted from greater *sl*NH_3_ accumulation due to its lower ASC (Fig. 7). This explanation is further supported by the static *nxr*A gene copies in soil *L* in contrast to substantial increases in *nxr*A observed in soil *W*.

Differences in ASC were related to differences in cation exchange capacity (CEC); i.e., the ratio (*W*:*L*) of *K*_*d*_ values in the two soils was 1.91 which was nearly identical to the ratio of their CEC values (1.93). Thus, while soil *L* and *W* had similar organic C and clay contents, differences in ASC were likely due to differences in chemical composition of soil organic matter and/or mineralogical composition of clay which control CEC^21^. It is not likely that a difference in the capacity of the two soils to fix N in clay minerals was an important factor; because NH_4_^+^ fixed by clay is not readily extracted by 2 M KCl nor is it readily available to microbes, such an effect would have been evident in differences by soil in *t*NH_4_^+^ that were not associated with differences in NAR and/or urea hydrolysis rates^22^.

Our calculations of *sl*NH_3_ concentrations are theoretical approximations. Because soil pH is by its nature operationally defined, any subsequent calculations are also operationally defined; e.g., a different range of absolute NH_3_ values would have resulted if a different pH solvent were used^23^, although *sl*NH_3_ levels based on a single solvent provide a basis for relative comparison. Our methods assumed that the NH_4_^+^-sorption equilibria in the isotherm experiments also described the solid-liquid NH_4_^+^ partitioning in the microcosm experiments. Nonetheless, it is interesting that the maximum *sl*NH_3_ concentrations observed in soil *L* (approximately 0.5 to 10 mg N L^−1^) were within the range observed to inhibit NOB and cause NO_2_^−^ accumulation in nitrifying wastewater systems^14^. Apart from any theoretical *sl*NH_3_ calculations, the multiple regression model (Fig. 6c) is further suggestive of NH_3_ toxicity; that is, increased *sl*NH_4_^+^ combined with reduced acidity (which together are the ‘raw ingredients’ for NH_3_ formation) explained 93% of the variance in *c-*NO_2_^−^.

The *sl*NH_3_ levels reached maximum values the day after N addition, but NO_2_^−^ did not reach maxima until at least Day 5 and generally remained elevated for longer than *sl*NH_3_. These results are reflected in stronger correlations between *c-*NH_3_ and *c-*NO_2_^−^ compared with correlations between point-in-time concentrations. This result could have been due to residual inhibitory effects of *sl*NH_3_ on NOB that persisted even after *sl*NH_3_ had subsided. It also is possible that once NO_2_^−^ started to accumulate, NO_2_^−^ itself (or its protonated form [HNO_2_]) became a source of toxicity to NOB. Kinetic models that account for NH_3_ and NO_2_^−^/HNO_2_ as separate sources of toxicity affecting AOB and NOB have been developed for wastewater systems^14^.

Previous studies have found a significant correlation between NO_2_^−^ and N_2_O dynamics^9^,^11^,^24^. The NO_2_^−^ molecule is an immediate precursor substrate for N_2_O produced via nitrifier-denitrification^19^ and chemo-denitrification^20^,^25^, and there is evidence that N_2_O can be produced via reaction of NO_2_^−^ with hydroxylamine (NH_2_OH)^26^. Previous studies have also found significant differences among soils in *p*N_2_O following amendment with NO_2_^−,^^20^,^27^. The current results show that *p*N_2_O is not necessarily a reliable indicator of actual N_2_O production (*a*N_2_O) following amendment of soil with BU or Ur; i.e., although soil *W* had greater *p*N_2_O after being artificially amended with NO_2_^−^, soil *L* had greater *a*N_2_O because it accumulated more NO_2_^−^ biologically than soil *W* following amendment with BU or Ur.

These experiments were not designed to precisely distinguish among all potential pathways of N_2_O production, e.g. nitrification, nitrifier-denitrification, heterotrophic denitrification, or chemodenitrification^19^,^20^,^28^,^29^. The experiments were designed to maintain aerobic conditions, and therefore the influence of denitrification of NO_3_^−^ as an N_2_O source was expected to be minimal. This was supported by the lack of positive correlation between *a*N_2_O and soil NO_3_^−^ levels; in fact, NO_3_^−^ was negatively correlated with *a*N_2_O in many cases. A previous study^20^ found that headspace O_2_ levels <5% were required for N_2_O production to proceed in NO_3_^−^-amended soil; in contrast, NO_2_^−^-amended soil readily produced N_2_O at ambient O_2_ and displayed increasing N_2_O per unit NO_2_^−^ as O_2_ decreased below 20%. Similar findings have been shown in culture studies examining nitrifier-denitrification^30^. Thus, in the current study, greater N_2_O production at greater water content was likely due to N_2_O derived from nitrifier-denitrification. At 100% of FC, nitrification proceeded more slowly than at 85% of FC, possibly due decreased O_2_ availability to support NH_4_^+^ oxidation. Because *t*NH_4_^+^ and pH did not decrease as quickly (owing to slower nitrification-induced H^+^ production), this resulted in greater *sl*NH_3_, which in turn could have caused the greater NO_2_^−^ at 100% of FC compared to 85% of FC. Thus, greater availability of NO_2_^−^, as well as increased potential for nitrifier-denitrification to produce N_2_O with reduced O_2_, likely enhanced N_2_O at 100% of FC.

Both soils responded differently to Ur compared to BU. With BU, it took 1 d for *t*NH_4_^+^ and pH to reach their maximum values compared to 5 d with Ur; this was likely due to compounds such as hippuric acid present in BU which accelerate Ur hydrolysis^31^. Soil pH also remained elevated for a longer period with Ur (again, indicating slower nitrification-induced H^+^ production), which resulted in a doubling of c-*sl*NH_3_ compared to BU. In soil *L*, this resulted in a 4- to 5-fold increase in NO_2_^−^ and a 7-fold increase in *c-*N_2_O. These results highlight the interactions involving several processes and substrates following BU and Ur addition that can regulate N_2_O production.

On the first day following addition of BU to soil *L* (in Series 1 and 2), N_2_O production was elevated without a corresponding increase in soil NO_2_^−^ or NO_3_^−^, and then declined on Day 3. This result was not observed with Ur, nor was it observed with soil *W*. Further research would be needed to explain this result, but it is possible that any NO_2_^−^ produced during the initial onset of nitrification was consumed in the N_2_O-producing reactions and therefore was not measurable. Urine addition could have stimulated ‘co-denitrification’ reactions^32^, or alternatively, N_2_O may have been produced from reactions involving NH_2_OH produced during AOB activity^28^.

While NH_3_ is the main substrate utilized by AOB^33^, at higher levels, NH_3_ can itself inhibit AOB activity. Decreasing NAR with increasing *sl*NH_3_ was observed in a grassland soil amended with BU and attributed to NH_3_ toxicity effects on AOB^34^. Similar results were found for NAR in Series 1 during Days 0–5. The qPCR data from Series 3 indicate that any NH_3_ toxicity effects on AOB were not large enough to inhibit *amo*A-b genes from increasing during Days 0–14. The *amo*A data also are consistent with results in New Zealand grasslands soils^35^, where *amo*A-b genes increased following N addition but *amo*A-a genes did not. Here, we found an increase in *amo*A-a numbers occurred only after *amo*A-b gene copies stopped increasing. This could indicate that *amo*A-b growth was inhibited after Day 14 by increasing acidity^36^ or NO_2_^−^/HNO_2_^14^ which might have favored *amo*A-a activity.

These results have implications for increased fundamental understanding as well as pointing in new directions for modeling and mitigating N_2_O emissions. The current experiments were aimed at studying processes occurring in localized zones receiving concentrated N inputs representative of potential N_2_O production hot spots. Under field conditions, these processes may be further modified by water infiltration or evaporation, temperature dynamics and other factors. Nonetheless, this study shows that soils having largely similar physical and chemical properties but differing in ASC can display dramatically different potentials for NH_3_ toxicity, NO_2_^−^ accumulation and N_2_O production. A previous study showed effects of altering CEC on nitrification dynamics^15^, but effects on NO_2_^−^ or N_2_O dynamics have not been considered. Further research comparing soils differing in ASC, and examining the effects of altering soil CEC, should be performed under both lab and field conditions to further investigate these variables as modeling parameters and potential N_2_O mitigation factors.

## Methods

### Soils

Soil ‘*L*’ was collected from a research field at Lincoln University, New Zealand (43.648 S; 172.454 E) that had been under pasture (*Lolium perenne* L.) for at least 5 yr. Soil ‘*W*’ was collected from a farmer’s field east of Waikari, New Zealand (42.964 S; 172.629 E) planted to alfalfa (*Medicago sativa* L.) and rotationally grazed by sheep (*Ovis aries*). Soils were collected from the upper 0.10 m and allowed to dry at 25 °C, and then ground and sieved (2 mm). Soils *L* and *W* were both classified as silt loams and had similar clay content (116 and 130 g kg^−1^, respectively), organic C (26, 33 g kg^−1^), C/N ratio (11.2, 10.6), and pH in H_2_O (6.3, 6.1), but differing CEC (14 and 27 cmol_c_ kg^−1^) (Table S6).

### Ammonium sorption capacity and solution-phase concentrations

Ammonium sorption isotherms were obtained using a batch equilibrium method^37^. Solutions (15 mL) containing NH_4_^+^ as NH_4_Cl (5, 10, 50, 100, 200, 300 and 400 mg NH_4_^+^-N L^−1^) were added to 50-mL polyethylene tubes containing 0.75 g soil. Three replicate tubes of each NH_4_^+^ concentration were equilibrated on a reciprocating shaker for 18 h at 100 rpm. Mixtures were filtered (Whatman 42) and the filtrate analyzed for NH_4_^+^ 38 with a flow-injection analyzer (FIA) (Lachat QuikChem 8500 or Alpkem FS3000). The amount of *sr*NH_4_^+^ was calculated from the difference in *sl*NH_4_^+^ at the beginning and end of equilibration and accounting for the initial 2 M KCl-extractable NH_4_^+^ content of the soil. The resulting data were not consistent (*R*^2^ < 0.6) with commonly used models^39^ but were well-described (*R*^2^ ≥ 0.99) by models of the form


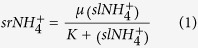


where μ (mg N kg^−1^) is the maximum sorption capacity and *K* (mg N L^−1^) is the *sl*NH_4_^+^ concentration at which *sr*NH_4_^+^ equals one-half of *μ*. Eq. (1) is similar in form to the Langmuir model and has a ‘linear portion’ for the case where *sl*NH_4_^+^<< *K,* such that *sr*NH_4_^+^=(*sl*NH_4_^+^) where *K*_*d*_ = *μ*/*K* is the slope with units (L kg^−1^). Eq. (1) was used to calculate theoretical concentrations of *sl*NH_4_^+^ and *sl*NH_3_ by expressing the total extractable ammonium (*t*NH_4_^+^, mg N kg^−1^) under equilibrium conditions as follows:


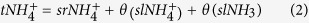


where θ is the soil water content (L kg^−1^). Eq. (1) was used to replace *sr*NH_4_^+^ in Eq. (2), and *sl*NH_3_ in Eq. (2) was replaced by:


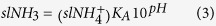


where *K*_*A*_ is the acid dissociation constant (9.245) for the NH_4_^+^-NH_3_ acid-base pair at 25 °C^40^. This resulted in the quadratic equation:


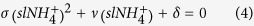


where σ, ν and δ are constants containing the parameters *μ*, *K*, θ, *K*_*A*_, *t*NH_4_^+^ and 10^pH^. For each sampling event, measured values of *t*NH_4_^+^ and pH were substituted into the constant terms, Eq. (4) was solved for *sl*NH_4_^+^ using the quadratic formula, and *sl*NH_3_ was calculated using Eq. (3) Example calculations are provided as Supplementary Information (SI). The above procedure was compared to another method where θ(*sl*NH_3_) was omitted from Eq. (2), and the results agreed within <1%. Thus, assumptions regarding whether *sl*NH_3_ was captured in the *t*NH_4_^+^ analysis affected the results to a negligible extent.

### Nitrite-addition experiments

The potential for each soil to produce N_2_O when amended with NO_2_^−^ was determined^20^. Soil was amended with solutions containing KNO_2_ to achieve concentrations of 0, 25, 50, 100 and 175 mg NO_2_^−^-N kg^−1^ at a water content equivalent to 85% of FC. Solutions were added to 10.0 g of air-dried soil in ‘wide-mouth’ 250 mL glass jars (69 mm diameter by 65 mm) and homogenized with a spatula. Jars were sealed with septum-equipped caps and incubated for 1 h at 25 °C with sampling of the headspace at 0, 30 and 60 min. Gas samples were immediately transferred to evacuated glass vials which were analyzed for N_2_O with a gas chromatograph (8610, SRI Instruments, CA) equipped with an electron capture detector and interfaced to an autosampler (Gilson 222XL, Middleton, WI)^41^. The rate of increase in headspace N_2_O concentration, headspace volume and soil mass were used to calculate *p*N_2_O^20^.

### Microcosm experiments

Three series of microcosm experiments were conducted. Series 1 used each soil amended with four rates of BU equivalent to 600, 800, 1000 and 1200 mg N kg^−1^ at a water content equivalent to 85% of FC. Series 2 used each soil amended with BU at 1000 mg N kg^−1^ at 100% of FC. Series 3 used each soil amended with Ur at 1000 mg N kg^−1^ at 85% of FC. At the start of the experiment, 21 replicate 250-mL glass jars of each treatment were established by adding solutions by pipette to 10.0 g of dry soil and homogenizing with a spatula such that the wetted soil occupied a thin layer (~3 mm) in the bottom of the jar. Three replicate jars of each treatment were sacrificed for destructive analysis after 1, 5, 8, 11, 14, 19 and 22 d. An additional three jars containing soil amended with deionized H_2_O were used to represent ‘Day 0’. Jars were incubated in the dark at 25 °C. On each sampling day, three randomly selected jars of each treatment were opened for 5 min to equilibrate the jar headspace with lab air and then sealed with caps, equipped with rubber septa, for 1 h. The jar headspace was sampled at 0, 30 and 60 min by syringe and *a*N_2_O was determined using methods described above. Nitrous oxide measured in the microcosm experiments is referred to as ‘actual’ N_2_O production rate (*a*N_2_O) to distinguish from *p*N_2_O. Immediately following gas sampling, approximately one-half of the soil mass in each jar was gravimetrically transferred to a polyethylene tube and extracted in 40 mL of 2 M KCl for 1 h. The extracts were filtered and stored at 4 °C until determination of *t*NH_4_^+,^^38^. Subsamples of the extract also were used to determine soil pH. Soil remaining in each jar was amended with 40 mL of a separate 2 M KCl solution and extracted for 10 min and then filtered for determination of NO_2_^−^ and the sum of NO_2_^−^+NO_3_^−^. The pH of the 2M KCl used for NO_2_^−^ and NO_2_^−^+NO_3_^−^ extraction was adjusted so that, during extraction, the pH of the soil-solution mixture was ≥ 8.5^42^. The NO_2_^−^ analysis was performed within 3 h with a spectrophotometer (Shimadzu UV mini–1240)^38^. The NO_2_^−^+NO_3_^−^ analysis was conducted within 24 h using a FIA preceded by Cd-reduction of NO_3_^−^ to NO_2_^−^, and NO_3_^−^ was determined by difference^38^.

The microcosms were designed to maintain aerobic conditions with minimal need for aeration due to the high ratio of headspace volume to soil mass and the high ratio of jar diameter to volume. The jars were opened for 10 min on Days 1, 8 and 15. This procedure minimized evaporative moisture losses while maintaining headspace O_2_ above 18% as determined by gas chromatographic analysis with a thermal conductivity detector. Opening of the jars on Day 1 also allowed release of CO_2_ produced during hydrolysis of Ur. Field capacities (0.35 and 0.45 kg H_2_O kg^−1^ for soil *L* and *W*, respectively) were determined by incremental water addition until free water was observed. Bovine urine was collected from the Lincoln University dairy farm where cows were grazing perennial ryegrass (*Lolium perenne* L.)/white clover (*Trifolium repens* L.). Urine was kept frozen until the day prior to setting up the experiment, at which time the urine was thawed and analyzed for total N content. Appropriate volumes of BU and H_2_O were added to soil in each jar to achieve target N concentrations and water contents. Because these grazed soils are commonly dry at the surface for days at a time prior to receiving urine deposition, we did not add water or pre-incubate the soils prior to amendment.

### Quantitative polymerase chain reaction (qPCR)

For Series 3, additional soil sub-samples were collected for DNA isolation and quantification of nitrifier gene abundances. On Days 0, 5, 8, 11, 14, 19, 22, 25 and 28, sub-samples (0.25 g) were extracted using a PowerLyzer PowerSoil DNA isolation Kit (MoBio, Carlsbad, CA) in accordance with manufacturer recommendations except for the final washing step which was performed twice rather than once. Abundances of 16S ribosomal RNA (16S rRNA), *amo*A-b, *amo*A-a and *nxr*A were determined using appropriate primers^43^,^44^,^45^,^46^. Abundances of *amo*A-b, *amo*A-a and *nxr*A were normalized to recovered 16S rRNA abundances^47^. Additional details are provided as SI.

### Data analysis

Concentrations of all N species and production of N_2_O are expressed on a dry weight soil basis. Chemical concentrations and pH determined at individual times were used to calculate cumulative ‘exposure’ indices using trapezoidal integration of concentration versus time data^7^,^9^,^48^. We use a ‘*c-*’ prefix to distinguish cumulative variables (e.g. *c-*NO_2_^−^) from point-in-time concentrations (e.g. NO_2_^−^). For cumulative acidity (*c-*H^+^), pH was first converted to theoretical hydrogen ion concentration using H^+^ = 10^−pH^ prior to integration. Integration of *a*N_2_O versus time also was performed, but in this case the resulting variable (*c-a*N_2_O) represents cumulative N_2_O production. The NAR was calculated over different time intervals from the difference in NO_2_^−^+NO_3_^−^ concentration divided by elapsed time. Three sets of data from the microcosm experiments were analyzed independently. The first set included all data from Series 1, the second set included data from Series 2 plus the 1000 mg N kg^−1^ treatment from Series 1 (to examine water content effects) and the third set included data from Series 3 plus the 1000 mg N kg^−1^ treatment from Series 1 (to examine N source effects). Each set was analyzed as a completely randomized design at *P* ≤ 0.05 using the MIXED procedure of SAS [Version 9.2, SAS Institute, Cary, NC] with time as a repeated measurement. Additional details are provided as SI.

## Additional Information

**How to cite this article**: Venterea, R. T. *et al.* Ammonium sorption and ammonia inhibition of nitrite-oxidizing bacteria explain contrasting soil N_2_O production. *Sci. Rep.* **5**, 12153; doi: 10.1038/srep12153 (2015).

## Supplementary Material

Supplementary Information

Supplementary Information

## Figures and Tables

**Table 1:** Cumulative indices for total extractable ammonium (*c-t*sNH_4_^+^), nitrite (*c-*NO_2_^−^), nitrate (*c-*NO_3_^−^), the sum of nitrite and nitrate (*c-* [NO_2_^−^ + NO_3 _^−^]), actual N_2_O production rate (*c-a*N_2_O), acidity (*c-*H^+^) and solution-phase ammonium (*c-sl*NH_4_^+^) and ammonia (*c-sl*NH_3_) as affected by soil type and rate of bovine urine (BU) in Series 1 experiments.

Soil	BU added (mg N kg^−1^)			
	600	800	1000	1200
*c*-*t*NH_4_^+^ (g N d kg^−1^)				
*L*	5.48 D‡	8.78 C	12.42 B	18.05 A
*W*	6.28 D	7.21 C	12.12 B	15.61 A
*P* > |*t*|	<0.001§	<0.001	0.216	<0.001
*c*-NO_2_^−^ (mg N d kg^−1^)				
*L*	23.95 D	59.55 C	209.37 B	761.04 A
*W*	15.43 B	18.72 B	72.64 A	94.38 A
*P* > |*t*|	0.005	<0.001	<0.001	<0.001
*c*-NO_3_^−^ (g N d kg^−1^)				
*L*	5.35 A	5.53 A	5.43 A	3.64 B
*W*	5.56 B	6.03 AB	6.22 AB	6.25 A
*P* > |*t*|	0.502	0.128	0.023	<0.001
*c*-(NO_2_^−^ + NO_3_^−^) (g N d kg^−1^)				
*L*	5.38 A	5.59 A	5.64 A	4.40 B
*W*	5.57 B	6.05AB	6.29 A	6.35 A
*P* > |*t*|	0.448	0.102	0.029	<0.001
*c*-H^+^ (mol H^+^ d kg^−1^)				
*L*	0.315 A	0.295 A	0.256 B	0.129 C
*W*	0.267 A	0.274 A	0.243AB	0.221 B
*P* > |*t*|	0.010	0.217	0.433	<0.001
*c*-aN_2_O (mg N_2_O-N kg^−1^)				
*L*	0.90 D	1.72 C	2.90 B	8.08 A
*W*	0.44 B	0.52 B	1.10 A	1.29 A
*P* > |*t*|	0.005	<0.001	<0.001	<0.001
*c*-*sl*NH_4_^+^ (g N d kg^−1^)				
*L*	0.94 D	2.02 C	4.45 B	10.29 A
*W*	0.35 C	0.41 C	0.74 B	1.01 A
*P* > |*t*|	0.020	<0.001	<0.001	<0.001
*c*-*sl*NH_3_ (mg N d kg^−1^)				
*L*	0.72 D	1.99 C	8.79 B	50.26 A
*W*	0.055 C	0.11 C	0.76 B	3.70 A
*P* > |*t*|	<0.001	<0.001	<0.001	<0.001

^‡^Within a row, means followed by the same letter are not significantly different at *P* ≤ 0.05.

^§^Significance of *t* test comparing the means from the two soils for a given rate of BU addition.
