# Corrigendum: Ammonium sorption and ammonia inhibition of nitrite-oxidizing bacteria explain contrasting soil N_2_O production

**DOI:** 10.1038/srep20720

**Published:** 2016-02-08

**Authors:** Rodney T. Venterea, Timothy J. Clough, Jeffrey A. Coulter, Florence Breuillin-Sessoms, Ping Wang, Michael J. Sadowsky

Scientific Reports 5: Article number: 1215310.1038/srep12153; published online: 07162015; updated: 02082016.

Ping Wang and Michael J. Sadowsky were omitted from the author list in the original version of this Article. This has been corrected in the PDF and HTML versions of the Article.

The Author Contributions section now reads:

“RV and TC designed and conducted the experiments. RV developed the equations, performed data reduction, prepared the figures and wrote the manuscript. JC performed the statistical analyses. PW, MS and FB-S developed the qPCR methods. FB-S conducted the qPCR analyses. TC, JC and FB-S revised and wrote specific sections of the manuscript.”

