# Supplementary material for: Ammonium sorption and ammonia inhibition of nitrite-oxidizing bacteria explain contrasting soil N2O production
Source: Sci Rep. 2015 Jul 16;5:12153. doi: 10.1038/srep12153 (PMC4503984; doi:10.1038/srep12153)
Supplement: Supplementary Information [file srep12153-s1.pdf]

Supplementary Information

Ammonium sorption and ammonia inhibition of nitrite-oxidizing bacteria explain contrasting soil N_2O production

*Rodney T. Venterea[1,2], Timothy J. Clough[3], Jeffrey A. Coulter[4], Florence Breuillin-Sessoms[5]

[1]USDA-ARS, Soil and Water Management Research Unit, St. Paul, MN 55108
[2]Dep. of Soil, Water, and Climate, Univ. of Minn., St. Paul, MN 55108
[3]Faculty of Agriculture and Life Science, Lincoln Univ., PO Box 85084, Lincoln 7647, Canterbury, New Zealand
[4]Dep. of Agronomy and Plant Genetics, Univ. of Minn., St. Paul, MN 55108
[5]Biotechnology Institute, College of Biological Sciences, Univ. of Minn., St. Paul, MN 55108
*Corresponding author: Phone: 612-624-7842, Email: Venterea@umn.edu

Supplementary Table S1. Nitrite+nitrate (NO_2^-+NO_3^-) accumulation rate (NAR) as affected by soil type and rate of bovine urine (BU) addition in Series 1 experiments†.

	BU added (mg N kg^{-1})			
Soil	600	800	1000	1200
	NAR (mg N kg^{-1} d^{-1})			
	Day 0-5			
L	11.08 A‡	9.78 A	6.82 B	3.78 C
W	23.96 A	21.95 A	15.64 B	12.42 C
$P > \|t\|$	§<0.001	<0.001	<0.001	<0.001
	Day 5-11			
L	31.14 A	29.74 A	26.62 A	20.67 B
W	27.92 B	33.49 AB	35.76 A	36.23 A
$P > \|t\|$	0.214	0.168	0.001	<0.001
	Day 11-19			
L	22.93 C	29.78 BC	39.39 A	34.40 AB
W	12.82 D	15.42 C	23.50 B	30.65 A
$P > \|t\|$	<0.001	<0.001	<0.001	0.239

† Statistical analysis is based on logarithm base 10 transformed data, and back-transformed means are reported.
‡ Within a row, means followed by the same letter are not significantly different at $P \leq 0.05$.
§ Significance of *t* test comparing the means from the two soils for a given rate of BU addition and time.

Supplementary Table S2. Cumulative indices for total extractable ammonium (c-tNH_4^+), the sum of nitrite and nitrate (c-[NO_2^-+NO_3^-]), N_2O production rate (c-aN_2O), and solution-phase ammonium (c-$slNH_4^+$) as affected by soil type and water content in incubation experiments following addition of 1000 mg N kg^{-1} of BU at 85 or 100% of field capacity (FC) in Series 2 experiments†.

	Water content (% of FC)	
Soil	100	85
	c-tNH_4^+ (g N d kg^{-1})	
L	13.16 A‡	12.43 B
W	14.03 A	12.12 B
$P > \|t\|$	0.002§	0.110
	c-(NO_2^- + NO_3^-) (g N d kg^{-1})	
L	4.86 B	5.64 A
W	4.92 B	6.29 A
$P > \|t\|$	0.669	0.006
	c-aN_2O (mg N_2O-N kg^{-1})	
L	14.86 A	2.91 B
W	2.71 A	1.11 B
$P > \|t\|$	<0.001	<0.001
	c-$slNH_4^+$ (g N d kg^{-1})	
L	4.36 A	4.43 A
W	0.88 A	0.74 B
$P > \|t\|$	<0.001	<0.001

† Statistical analyses are based on logarithm base 10 transformed data, and back-transformed means are reported.
‡ Within a row, means followed by the same letter are not significantly different at $P \leq 0.05$.
§ Significance of *t* test comparing the means from the two soils for a given water content.

Supplementary Table S3. Cumulative indices for extractable nitrite (c-NO_2^-) and nitrate (c-NO_3^-), acidity (c-H^+) and solution-phase ammonia (c-$slNH_3$) as affected by soil type and water content in incubation experiments following addition of 1000 mg N kg^{-1} of bovine urine at 85 or 100% of field capacity (FC) in Series 2 experiments†.

	c-NO_2^- (g N d kg^{-1})	c-NO_3^- (g N d kg^{-1})	c-H^+ (mol H^+ d kg^{-1})	c-$slNH_3$ (mg N d kg^{-1})
Soil				
L	246	5.00	0.195	15.2
W	81.5	5.53	0.194	2.85
$P > \|t\|$	<0.001‡	0.002	0.951	<0.001
Water content (% of FC)				
100	187	4.70	0.139	13.3
85	141	5.83	0.250	4.77
$P > \|t\|$	0.011	<0.001	<0.001	<0.001

† Statistical analyses are based on logarithm base 10 transformed data, and back-transformed means are reported.
‡ Significance of *t* test comparing the means from the two soils for a given water content.

Supplementary Table S4. Cumulative indices for total extractable ammonium (c-$t\mathrm{NH_4^+}$), nitrite (c-$\mathrm{NO_2^-}$), nitrate (c-$\mathrm{NO_3^-}$), actual N_2O production rate (c-$a\mathrm{N_2O}$), acidity (c-$\mathrm{H^+}$) and solution-phase ammonium (c-$sl\mathrm{NH_4^+}$) and ammonia (c-$sl\mathrm{NH_3}$) as affected by soil type and N source in incubation experiments following addition of 1000 mg N kg^{-1} of bovine urine (BU) or urea (Ur) at 85% of field capacity in Series 3 experiments†.

	N source	
Soil	BU	Ur
	c-$t\mathrm{NH_4^+}$ (g N d kg^{-1})	
L	12.42 B‡	15.69 A
W	12.12 B	12.84 A
$P > \|t\|$	0.186§	<0.001
	c-$\mathrm{NO_2^-}$ (mg N d kg^{-1})	
L	209 B	1213 A
W	72.6 A	40.4 B
$P > \|t\|$	<0.001	<0.001
	c-$\mathrm{NO_3^-}$ (g N d kg^{-1})	
L	5.43 A	3.76 B
W	6.22 A	6.18 A
$P > \|t\|$	0.053	<0.001
	c-$a\mathrm{N_2O}$ (mg N_2O-N kg^{-1})	
L	2.91 B	20.91 A
W	1.11 B	1.95 A
$P > \|t\|$	<0.001	<0.001
	c-$\mathrm{H^+}$ (mol H^+ kg^{-1})	
L	0.256 A	0.039 B
W	0.243 A	0.164 B
$P > \|t\|$	0.232	<0.001
	c-$sl\mathrm{NH_4^+}$ (g N d kg^{-1})	
L	4.45 B	6.91 A
W	0.74 A	0.80 A
$P > \|t\|$	<0.001	<0.001
	c-$sl\mathrm{NH_3}$ (mg N d kg^{-1})	
L	8.78 B	19.44 A
W	0.76 B	1.35 A
$P > \|t\|$	<0.001	<0.001

† Statistical analyses are based on logarithm base 10 transformed data, and back-transformed means are reported.
‡ Within a row, means followed by the same letter are not significantly different at $P \leq 0.05$.
§ Significance of *t* test comparing the means from the two soils for a given N source.

Supplementary Table S5. Pearson's correlation coefficient (*r*) for actual N_2O production rate (aN_2O) versus soil nitrite (NO_2^-), nitrate (NO_3^-) and the sum of ($NO_2^- + NO_3^-$), and for soil NO_2^- versus soil total extractable ammonium (*t*NH_4^+), solution-phase ammonium (*sl*NH_4^+) and ammonia (*sl*NH_3) for soils treated with bovine urine (BU) or urea (Ur). Cumulative indices are denoted by '*c*-'†.

Dependent variable	Micro-cosm series	N Source	Field capacity (%)	*n*	Independent variable		
					NO_2^-	NO_3^-	NO_2^-+NO_3^-
aN_2O	1	BU	85	168	0.76***	-0.58***	-0.51***
	2	BU	100	42	0.58***	0.12ns	0.14ns
	3	Ur	85	42	0.92**	0.35*	0.46**
	1,2	BU	85, 100	210	0.70***	-0.41***	-0.36***
	1,3	BU, Ur	85	210	0.83***	-0.32***	-0.24***
	1,2,3	BU, Ur	85, 100	252	0.78***	-0.26***	-0.19**
					c-NO_2^-	*c*-NO_3^-	*c*-(NO_2^-+NO_3^-)
c-*a*N_2O	1	BU	85	24	0.95***	-0.80***	-0.69***
	2	BU	100	6	0.96**	-0.70ns	-0.19ns
	3	Ur	85	6	0.99***	-0.96**	-0.93**
	1,2	BU	85, 100	30	0.87***	-0.74***	-0.69***
	1,3	BU, Ur	85	30	0.94***	-0.83***	-0.64***
	1,2,3	BU, Ur	85, 100	36	0.91***	-0.80***	-0.66***
					tNH_4^+	*sl*NH_4^+	*sl*NH_3
NO_2^-	1	BU	85	192	0.59***	0.73***	0.31***
	2	BU	100	48	0.50***	0.58***	0.30*
	3	Ur	85	48	0.49***	0.75***	0.18ns
	1,2	BU	85, 100	240	0.57***	0.70***	0.32***
	1,3	BU, Ur	85	240	0.55***	0.72***	0.27***
	1,2,3	BU, Ur	85, 100	288	0.54***	0.70***	0.28***
					c-*t*NH_4^+	*c*-*sl*NH_4^+	*c*-*sl*NH_3
c-NO_2^-	1	BU	85	24	0.88***	0.92***	0.96***
	2	BU	100	6	-0.99***	0.98***	0.94**
	3	Ur	85	6	0.99***	0.99***	0.99***
	1,2	BU	85, 100	30	0.86***	0.91***	0.95***
	1,3	BU, Ur	85	30	0.82***	0.93***	0.94***
	1,2,3	BU, Ur	85, 100	36	0.80***	0.92***	0.93***

†All variables were logarithm base 10 transformed prior to analysis.

‡ ns, not significant.

* $P < 0.05$. ** $P < 0.01$. *** $P < 0.001$.

Supplementary Methods

Supplementary Table S6. Selected properties of soils *L* and *W*.

Property	Unit	Soil	
		L	*W*
Textural class (USDA)	---	silt loam	silt loam
Sand (> 63μm)	$g\ kg^{-1}$	380	304
Silt (2-63μm)		504	566
Clay (< 2μm)		116	130
Organic C	$g\ C\ kg^{-1}$	25.5	33.0
C/N ratio	$kg\ C\ kg\ N^{-1}$	11.2	10.6
pH (water)	----	6.3	6.1
pH (1 M KCl)	----	5.4	5.4
Cation exchange capacity	$cmol_c\ kg^{-1}$	14	27

qPCR

Total DNA was quantified spectrophotometrically with a Qubit 2.0 Fluorometer (Thermo Fisher Scientific, Waltham, MA). Total DNA was diluted to a concentration of 10 ng μL^{-1} followed by qPCR using a LightCycler 480 System (Roche Diagnostics, Indianapolis, IN) with the iTaq Universal SYBR Green Supermix (Biorad, Hercules, CA). Each qPCR reaction was performed in triplicate in a 20 μL volume containing 10 μL of SYBR green mix, 0.8 μL of each primer (10 mM), 5 μL of diluted DNA and 3.4 μL of DNAse free H_2O. Thermocycling was performed using the conditions described in Table S8. Primer efficiency was calculated for each pair of primers (Table S9). The number of gene copies was calculated using the standard curve method and the specificity of the primers was determined by agarose gel electrophoresis.

Supplementary Table S7. Primers used in this study.

	Primer	Amplicon length (bp)	Reference
16S rRNA	U515F: 5'-GTGCCAGCMGCCGCGGTA-3'<br>U806R: 5'-GGACTACHVGGGTWTCTAAT-3'	292	S1
*amo*A-a	amoA-AF: 5'-STAATGGTCTGGCTTAGACG-3'<br>amoA-AR: 5'-GCGGCCATCCATCTGTATGT-3'	635	S2
*amo*A-*b*	amoA-1F: 5'GGGGTTTCTACTGGTGGT-3'<br>amoA-2R 5'-CCCCTCKGSAAAGCCTTCTTC-3'	491	S3
*nxr*A	F1norA: 5'-CAGACCGACGTGTGCGAAAG-3'<br>R2norA: 5'-TCYACAAGGAACGGAAGGTC-3'	323	S4

Supplementary Table S8. PCR conditions for primers.†

Step		Time (s)			
	Temperature (°C)	16S rRNA	*amo*A-a	*amo*A-*b*	*nxr*A
Denaturation (initial)	95	300	300	300	300
Denaturation	95	30	45	45	30
Annealing	50 (16S rRNA)<br>57 (*amo*A-*b*)<br>55 (*amo*A-a and *nxr*A)	30	45	45	30
Extension	72	30	60	60	45
Dissociation stage	60-95	5	5	5	5

† Each qPCR was run for 45 cycles.

Supplementary Table S9. Validation of qPCR.

Parameter	16S rRNA	*amo*A-a	*amo*A-*b*	*nxr*A
†Calibration (copies/µl)	3.10^1-3.10^5	3.10^2-3.10^5	3.10^2-3.10^5	3.10^1-3.10^5
R^2	0.9972	0.9996	0.9999	0.9998
‡Efficiency (%)	99	91	89	92

† Standard template dilution was used to create the standard curve for each pair of primers, and primer efficiency and gene copy number were calculated based on the standard curve.
‡ Primer efficiency (E) was calculated using the slope obtained from the standard curve as $E = (10^{(-1/slope)}-1)*100$.

Data analysis

Soil type, N rate, water level, N source and time were considered fixed effects, and replication and interactions with replication were considered random effects. The first-order autoregressive covariance structure was selected based on the Akaike's and Schwarz' Bayesian criteria (Reference S5). Residuals were inspected for normality and common variance using the UNIVARIATE procedure of SAS and scatterplots of residuals vs. predicted values (Reference S6). Assumptions of normality and common variance were not met for any dependent variables except pH, so these variables were log base 10 transformed prior to statistical analysis. Mean comparisons were made using independent pairwise *t* tests ($P \leq 0.05$) using the PDIFF option in the MIXED procedure of SAS. Linear associations between variables were evaluated with Pearson's correlation coefficient at $P \leq 0.05$ using the CORR procedure of SAS. Linear regression equations were developed to describe relationships between variables using the REG procedure of SAS [Version 9.2, SAS Institute, Cary, NC]. Nonlinear regression equations were developed to describe the responses of $srNH_4^+$ to $slNH_4^+$ in the sorption experiment and pN_2O to NO_2^- in the nitrite addition experiment using the NLIN procedure of SAS. The reported regression models were selected based on scatterplots of residuals vs. predicted values (Reference S6) and were significant at $P \leq 0.001$. Linear multiple regression models with c-NO_2^- or c-aN_2O as the dependent variable and c-$slNH_4^+$ and c-H^+ as independent predictors were evaluated for all microcosm data (Series 1-3) using the REG procedure of SAS. Reported regression models and all parameter estimates were significant at $P < 0.01$.

Supplementary References

S1. Caporaso, J.G. et al. Ultra-high-throughput microbial community analysis on the Illumina HiSeq and MiSeq platforms. *ISME J.* **6**, 1621–1624 (2012).

S2. Francis, C.A., Roberts, K.J, Beman, J.M., Santoro, A.E. & Oakley, B.B. Ubiquity and diversity of ammonia-oxidizing archaea in water columns and sediments of the ocean. *P. Natl. Acad. Sci. USA*. **102**, 14683–14688 (2005).

S3. Rotthauwe, J.H., Witzel, K.P. & Liesack, W. The ammonia monooxygenase structural gene amoA as a functional marker: Molecular fine-scale analysis of natural ammonia-oxidizing populations. *Appl. Environ. Microbiol*. **63**, 4704-4712 (1997).

S4. Wertz, S., Poly, F., Le Roux, X. & Degrange, V. Development and application of a PCR-denaturing gradient gel electrophoresis tool to study the diversity of *Nitrobacter*-like nxrA sequences in soil. *FEMS Microbiol. Ecol*. **63**, 261 (2008).S3. Francis et al., 2005

S5. Littell, R.C. et al. in *SAS for mixed models 2nd edn,* (SAS Institute, 2006).

S6. Kutner, M.H., Nachtsheim, C.J. & Neter, J. *Applied linear regression models.* 4th ed. McGraw-Hill, New York (2004).
